# Intrinsic ankle stiffness is associated with paradoxical calf muscle movement but not postural sway or age

**DOI:** 10.1113/EP091660

**Published:** 2024-03-15

**Authors:** Raymond F. Reynolds, Anna M. Liedtke, Martin Lakie

**Affiliations:** ^1^ School of Sport, Exercise & Rehabilitation Sciences University of Birmingham Birmingham UK

**Keywords:** ankle stiffness, muscle movement, postural control, ultrasound

## Abstract

Due to Achilles tendon compliance, passive ankle stiffness is insufficient to stabilise the body when standing. This results in ‘paradoxical’ muscle movement, whereby calf muscles tend to shorten during forward body sway. Natural variation in stiffness may affect this movement. This may have consequences for postural control, with compliant ankles placing greater reliance upon active neural control rather than stretch reflexes. Previous research also suggests ageing reduces ankle stiffness, possibly contributing to reduced postural stability. Here we determine the relationship between ankle stiffness and calf muscle movement during standing, and whether this is associated with postural stability or age. Passive ankle stiffness was measured during quiet stance in 40 healthy volunteers ranging from 18 to 88 years of age. Medial gastrocnemius muscle length was also recorded using ultrasound. We found a significant inverse relationship between ankle stiffness and paradoxical muscle movement, that is, more compliant ankles were associated with greater muscle shortening during forward sway (*r* ≥ 0.33). This was seen during both quiet stance as well as voluntary sway. However, we found no significant effects of age upon stiffness, paradoxical motion or postural sway. Furthermore, neither paradoxical muscle motion nor ankle stiffness was associated with postural sway. These results show that natural variation in ankle stiffness alters the extent of paradoxical calf muscle movement during stance. However, the absence of a clear relationship to postural sway suggests that neural control mechanisms are more than capable of compensating for a lack of inherent joint stiffness.

## Abstract (highlights)

- **What is the central question of this study?**
  Do individual variations in standing ankle stiffness affect calf muscle movement, and is this related to postural sway or age?
- **What is the main finding and its importance?**
  Less stiff ankles are associated with a greater tendency for ‘paradoxical’ calf muscle movement during standing, whereby the muscle shortens during forward sway. However, this tendency was not related to postural sway or age. This suggests that neural control mechanisms are more than capable of compensating for a lack of inherent joint stiffness.

## 1 INTRODUCTION

During quiet stance the body centre of mass lies just forward of the ankle joint, and the calf muscles must generate continuous ankle torque in order to prevent the body toppling forwards (Lakie & Loram, 2004). However, it is generally not possible to achieve stability simply by maintaining a fixed level of calf muscle activity. Active modulation of muscle length is necessary. This is because the stiffness of the ankle joint is insufficient to resist the gravitational toppling torque of the body. Estimates of passive ankle stiffness during standing vary from approximately 30% to 90% of the body's gravitational toppling torque (Casadio et al., 2005; Loram & Lakie, 2002; Sakanaka et al., 2015). This low joint stiffness is attributable to the most elastic in‐series structure, namely, the Achilles tendon. The Achilles is highly compliant during the relatively low ankle torque experienced during quiet stance. One consequence of this compliance is that the calf muscles tend to move in a ‘paradoxical’ manner. That is to say, as the body sways forwards (backwards) the calf muscles tend to shorten (lengthen). This has been clearly demonstrated with the use of ultrasound muscle tracking (Fitzpatrick & Gandevia, 2005; Loram et al., 2004, 2005, 2009).

Paradoxical calf muscle motion has a number of implications for the control of standing. Firstly, simple spinal stretch reflexes have minimal role in quiet standing since the calf muscles are not lengthening during forward sway. Secondly, anticipatory muscle control must be necessary for stability. This renders the control task more difficult from a neural control perspective (Lakie & Loram, 2004). Lastly, calf muscle length cannot encode body position in a simple and direct way, since muscle movement and body sway are uncoupled (Di Giulio et al., 2009). All of these factors may have consequences for postural stability. Hence, greater intrinsic ankle stiffness may confer an advantage in terms of muscle movement, potentially leading to greater postural stability.

Previous research suggests that natural variations in passive ankle stiffness may influence paradoxical muscle motion. Lakie et al. (2003) studied a task analogous to standing, involving manual balancing of an inverted pendulum. They systematically varied the compliance of the linkage between the hand and pendulum, and found that this modulated the kinematic relationship between the two. With a stiff linkage, the hand and pendulum moved as one (‘orthodoxical’). But when stiffness dropped below the critical load stiffness of the pendulum, the hand and pendulum tended to move in opposite directions (‘paradoxical’). In terms of real standing, considerable variation in passive ankle stiffness has been demonstrated between people. Similar to the manual pendulum task, such variation may affect the extent of paradoxical calf muscle movement. This, in turn, would affect postural control. For example, a person with a very stiff ankle joint may exhibit greater ‘orthodox’ muscle movement. This might allow them to place more reliance upon simple stretch reflexes and require less active neural control from supraspinal areas. It may also improve the quality of proprioceptive information from the calf muscle, since muscle length changes will be more closely coupled to body position. This may benefit postural stability. Here we directly test the hypothesis that natural variations in passive ankle stiffness affect the degree of paradoxical muscle movement during quiet standing. Specifically, we predict that those with more compliant ankles should exhibit a stronger inverse relationship between muscle length and body position, and exhibit greater postural sway.

Ageing may affect this relationship via changes in ankle stiffness. As stated above, the limiting factor in overall ankle stiffness during standing is the Achilles tendon (Loram & Lakie, 2002). Tendon stiffness generally matches the size and strength of its attached muscle (Muraoka et al., 2005). Changes in muscle mass due to training or inactivity have been associated with concomitant increases and decreases in tendon stiffness, respectively (Morrissey et al., 2011). Since ageing is associated with muscle loss, one might therefore expect reduced Achilles tendon stiffness, as well as an overall reduction in ankle stiffness. Onambele et al. (2006) have indeed demonstrated reduced Achilles tendon stiffness with age, but Chesworth and Vandervoort (1989) showed no age‐related changes in overall ankle stiffness. However, in these papers (and others), stiffness was assessed while seated or prone. To our knowledge, no one has investigated the effect of age upon *functional* ankle stiffness, as measured during quiet stance. Joint stiffness is known to be affected by various factors, including joint angle and muscle activity (Mirbagheri et al., 2000). To determine the functional role of ankle stiffness in standing, it is therefore important to measure it during standing conditions. Here we achieve this using a motorised horizontal platform capable of rotating around the ankle joint axis. We hypothesise that normal ageing will be associated with reduced standing ankle stiffness, and that this will increase the extent of paradoxical muscle movement, with potential consequences for postural stability.

We address the above hypotheses by measuring calf muscle motion during standing with ultrasound, in addition to measuring ankle stiffness with the use of support‐surface perturbations. To determine effects of age, we performed these tests in 40 adults ranging from 18 to 88 years of age.

## 2 METHODS

### 2.1 Ethical approval

This research was conducted in accordance with the *Declaration of Helsinki*, except for registration in a database. Ethical Approval was granted from the University of Birmingham STEM ethics committee (ERN_15‐0674). All participants gave written informed consent.

### 2.2 Participants

Forty volunteers between the ages of 18 and 88 participated in this research (21 males, 19 females; (means ± SD) age: 54.1 ± 24.1 years; height: 1.71 ± 0.11 m; mass: 68.2 ± 13.6 kg). All subjects were healthy, with no known neurological illness or musculoskeletal injuries which might affect standing.

### 2.3 Procedure and apparatus

Data collection for each subject consisted of two parts. The first part involved the use of small perturbations to estimate ankle stiffness while subjects stood quietly. The second part involved no perturbations and was used to record muscle movement and postural sway during quiet stance and voluntary sway. All conditions were performed with the eyes open.

#### 2.3.1 Ankle stiffness

**FIGURE 1:**
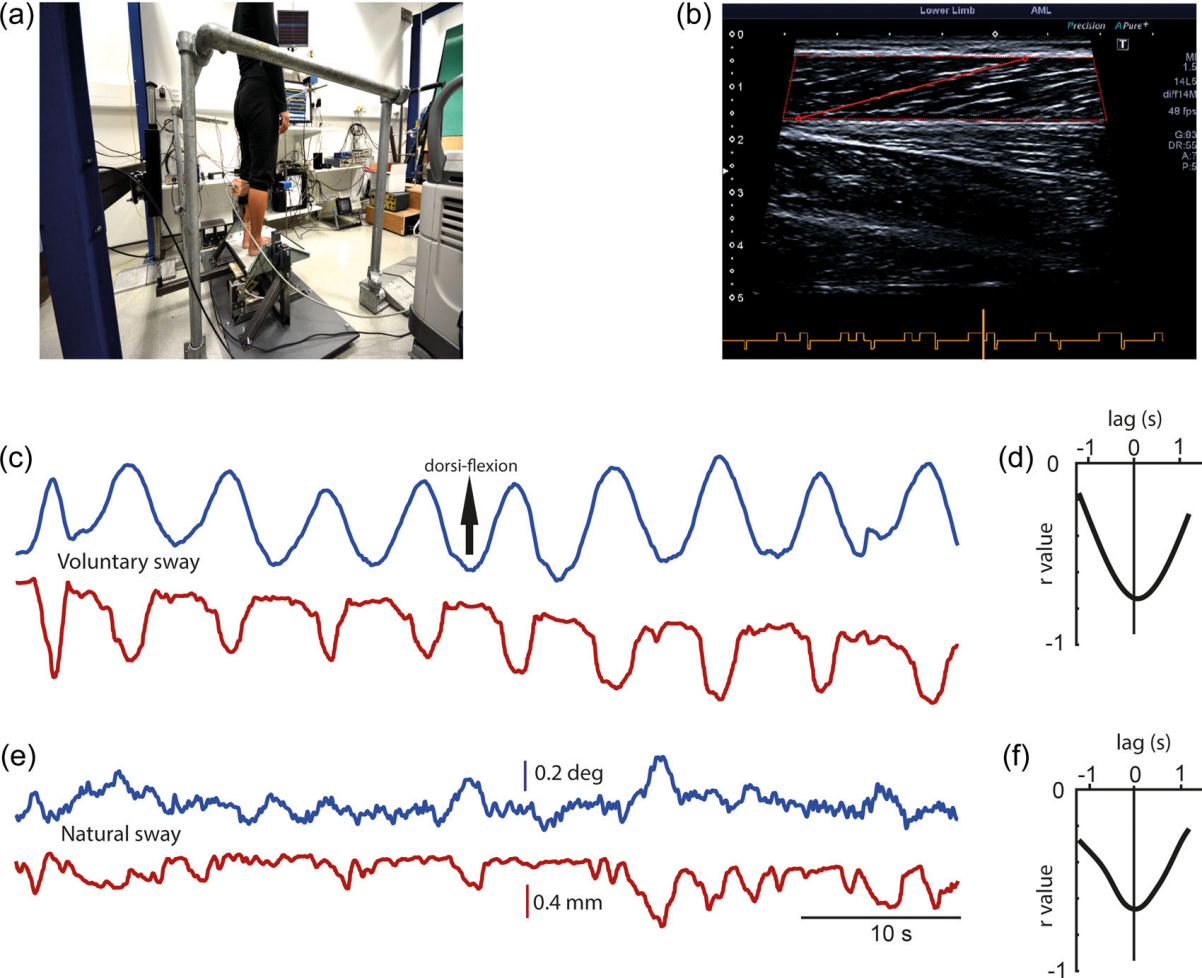
Ultrasound tracking of calf muscles during standing. (a) A subject standing on the perturbation platform with the ultrasound probe attached to their left medial gastrocnemius. (b) The resulting ultrasound image, with red lines depicting the MATLAB‐tracked fascicle length. (c) Ankle angle (blue) and muscle fascicle length (red) during a period of voluntary sway in the antero‐posterior plane. (d) The cross‐correlation between ankle angle and muscle length. The strong negative correlation represents ‘paradoxical’ muscle movement, that is, during forward sway the muscle shortens. (e, f) The same measurements as in (c) & (d) for a period of normal quiet standing.

**FIGURE 2:**
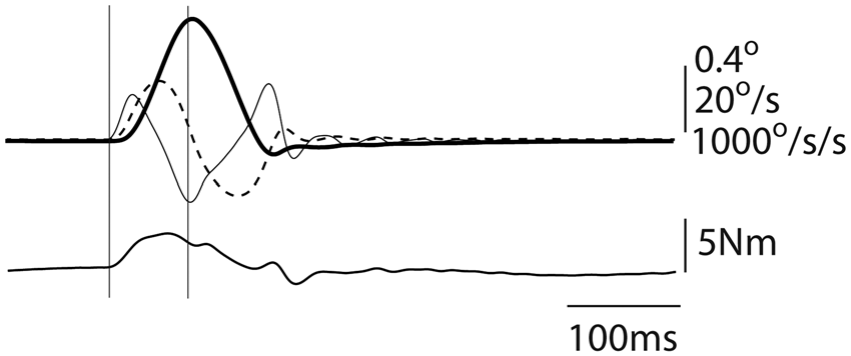
Perturbation for assessing ankle stiffness. Platform kinematics and torque signals are shown for a single 0.8° toes‐up perturbation. The upper panel shows position (thick continuous line), velocity (dashed line) and acceleration (thin continuous line). The lower panel shows torque. Stiffness was calculated from a 70 ms time window shown by the vertical lines.

Standing ankle stiffness was determined using a previously published method (Sakanaka et al., 2015). Participants stood on a custom‐built motorised platform that could rotate about an axis 8.5 cm above platform height, co‐linear with the ankle joint (see Figure 1a). A linear servo motor (XTA3808S; Dunkermotoren, Nonndorf, Germany) drove the platform via a lever positioned perpendicular to the platform. The motor operated in position control mode in order to drive the footplate to specified angles irrespective of resistance offered by the equipment or subject. Participants stood with both feet on the same platform with the feet no closer together than 8 cm and no further apart than hip width. Care was taken to ensure that the ankle joint was aligned with the axis of rotation. Small, brief platform perturbations were applied in both toes‐up and ‐down directions. Since joint stiffness has been shown to depend upon perturbation amplitude (Kearney & Hunter, 1982; Sakanaka et al., 2015), we applied two different perturbations of 0.2° and 0.8°, each consisting of a raised cosine waveform lasting 140 ms. These two values were chosen to span the thixotropic range of the muscle (Sakanaka et al., 2015). A representative example of kinematic and torque traces can be seen in Figure 2 for a 0.8° perturbation. Perturbations were applied in a random order every 4–5 s while participants stood quietly for trials lasting 1 min. Twelve perturbations were applied during each trial, comprising three repeats of each condition (0.8, 0.2, toes‐up, toes‐down). The torque response to each perturbation was measured using a load cell located underneath the platform situated in‐series between the driving lever and the platform itself (F256, Novatech Measurements Ltd, St Leonards on Sea, UK). The load cell was oriented horizontally and completely uncoupled from vertical loads. Hence, it was unaffected by the mass of the person standing on the platform, and only transduced forces applied distal to the platform axis (i.e., it only responded to ankle torque). Platform position was measured using an incremental rotary encoder in a 7‐1 gearing (Hengstler, Aldingen, Germany). All signals were acquired at 1 kHz by an NI‐PCI‐6229 data acquisition card using Simulink Desktop Real‐Time (The MathWorks, Natick, MA, USA).

#### 2.3.2 Muscle movement and postural sway

Once the platform perturbations were completed the ultrasound recordings were performed. Loram et al. (2004) measured the correlation between muscle length and body position during slow voluntary sway. We chose to replicate this condition, and also included a condition of normal quiet standing to ensure that the findings extend to involuntary postural control. Two laser reflex sensors were used to measure horizontal translation of the body in the antero‐posterior plane at the level of the umbilicus and the dorsal surface of the left tibia approximately half way up the lower leg (Model YT44MGV80; Wenglor Sensoric, Tettnang, Germany). These sensors work by measuring the angle of a reflected laser beam and have a resolution of ∼0.2 mm. The umbilical signal was used to measure body sway, while the tibial signal was used for cross‐correlation of ankle angle with muscle length. During voluntary sway, participants were provided with visual feedback of the umbilical laser sensor and asked to track a moving target at 0.2 Hz, corresponding to a peak body motion of 1.5°. During quiet standing, participants were simply told to stand still but relaxed. Movement of the left medial gastrocnemius muscle was recorded using a linear ultrasound transducer (PLT‐1005BT; 10 MHz, 58 mm field of view width, connected to Aplio 500 ultrasound machine; Toshiba, Tokyo, Japan). The transducer was attached to the surface of the calf at the level of the muscle belly, oriented in a superior–inferior direction. Care was taken to ensure sufficient slack in the ultrasound cable in order to preclude sway feedback. A total of 12 1‐min trials were recorded, six for quiet stance and six for voluntary sway. Ultrasound video was streamed from the Aplio 500 DVI port into Simulink at 50 Hz using a PCIe video capture card (Startech, London, Canada). Simulink allowed for ultrasound images to be synchronised with simultaneously recorded sway signals.

### 2.4 Data analysis

#### 2.4.1 Ankle stiffness

All data analysis was performed using MATLAB (The MathWorks). Ankle stiffness was estimated using a previously validated technique (Loram & Lakie, 2002; Sakanaka et al., 2015). First, platform position was differentiated twice using the Savitzky‐Golay filter technique to obtain velocity and acceleration. Regression was then applied to these time series traces, in order to derive stiffness, viscosity and inertia, according to the following equation:

$$T\;=\;K\theta +B\dot{\theta }+I\ddot{\theta }$$

where: *T* is torque, θ is ankle angle, *K* is stiffness, $\dot{\theta }$ is angular velocity, *B* is viscosity, $\ddot{\theta }$ is angular acceleration and *I* is the moment of inertia of the foot and platform combined. This analysis was confined to the first 70 ms of the perturbation to minimise any reflex contributions to ankle torque. Consistent with previous research (Sakanaka et al., 2015, 2021), there was no effect of perturbation direction upon ankle stiffness estimates (5.49 ± 1.21 N m/deg (toes up); 5.51 ± 1.06 N m/deg (toes down); *t* = 0.21, *P* = 0.835). Therefore, both directions were combined for all further analysis.

In addition to estimating absolute stiffness, we also expressed ankle stiffness as a percentage of gravitational toppling torque per unit angle, for each person. This provides a functional measure of ankle stiffness, allowing for comparison between participants despite differences in mass and height. Toppling torque was calculated by obtaining a linear fit between ankle torque and body angle during a period of quiet stance, and is expressed in units of N m/deg. When ankle stiffness is then expressed as a percentage of this toppling torque, we refer to this as relative stiffness, labelled as ‘% TT’.

#### 2.4.2 Postural sway

All postural sway measurements reported here are those recorded during the quiet standing condition. The position signal recorded from the umbilical laser reflex sensor was trigonometrically converted to provide a measure of antero‐posterior body angle. This was done by taking the inverse tangent of the ratio of the position signal and the height of the laser above the ankle joint. After applying a 5 Hz lowpass butterworth filter, this signal was then differentiated to provide body angular velocity. Postural sway was then taken as the root‐mean‐square value of this velocity signal. The same trigonometric calculation was also performed for the ankle laser sensor to provide ankle angular position.

#### 2.4.3 Muscle movement

Ultrasound images were tracked using a previously published and validated software tracking algorithm designed to track the gastrocnemius muscle (Cronin et al., 2011). This provided fascicle length (FL) and pennation angle (PA) of the medial gastrocnemius. Changes in muscle length (ML) were calculated from these parameters as follows:

$$\mathrm{ML}=\left(\mathrm{FL}\times \cos\left(\mathrm{PA}\right)\right)-\mathrm{mean}\left(\mathrm{FL}\times \cos\left(\mathrm{PA}\right)\right)$$

The relationship between muscle length and ankle angle was then determined by normalised cross‐correlation (Figure 1). Values varied between +1 and −1, representing perfect positive and inverse correlations, respectively. Negative correlation values are indicative of paradoxical muscle movement, that is, muscle shortening during forward sway. For each person we took the nadir value for comparison against ankle stiffness.

#### 2.4.4 Statistics

To determine effects of age, comparisons were performed between participants over and below 50 years of age (*n* = 24 and 16, respectively). Repeated‐measures ANOVA was used to determine effects of age and perturbation amplitude upon absolute and relative ankle stiffness. The Mann–Whitney test was used to compare sway velocity between young and old, since these data were not normally distributed. Pearson's correlation was used to determine the relationship between relative ankle stiffness and the amplitude of muscle length–sway angle cross‐correlation. Data are presented as mean ± 1 standard deviation unless otherwise stated. *P* < 0.05 was considered significant for all statistical tests.

## 3 RESULTS

### 3.1 Postural sway and ankle stiffness

**FIGURE 3:**
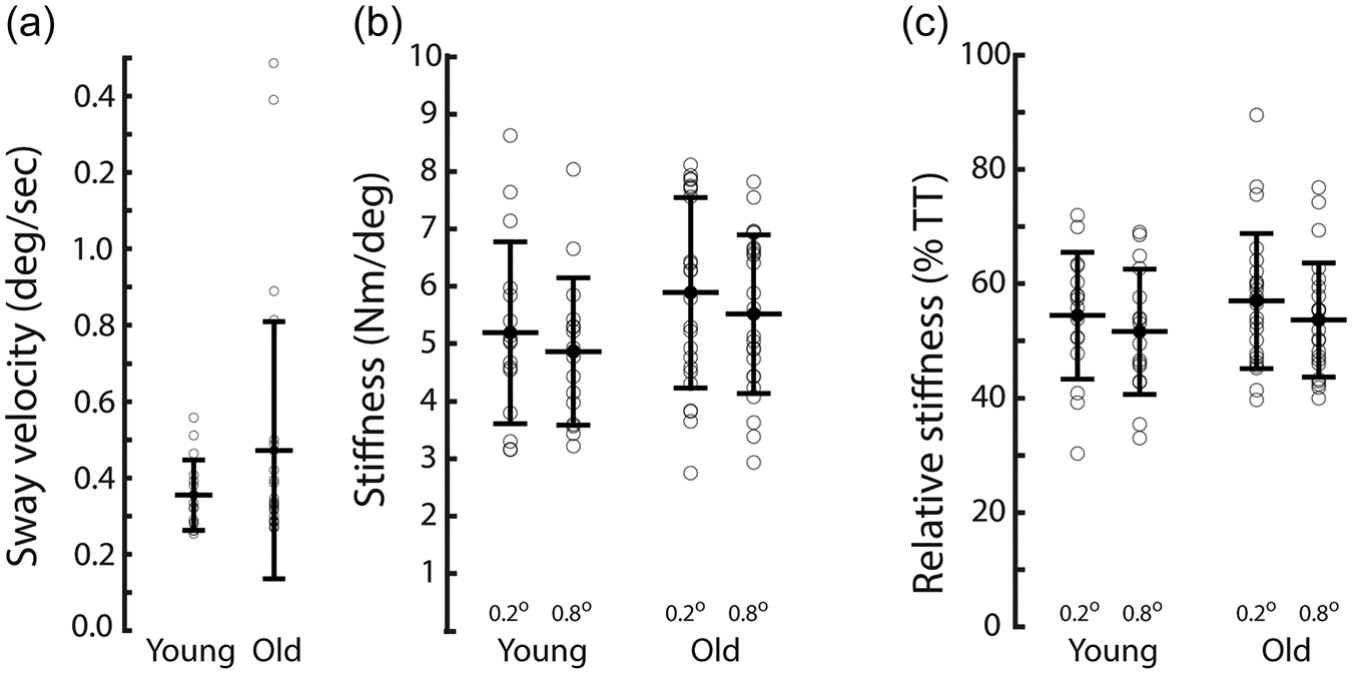
Sway and ankle stiffness across age groups. (a) Root‐mean‐square antero‐posterior sway velocity is shown for young (*n* = 16) and old (*n* = 24). (b) Ankle stiffness is shown separately for 0.2° and 0.8° perturbations. (c) Stiffness expressed as a percentage of gravitational toppling torque. Mean values ± 1 standard deviation are shown by horizontal lines. Individual participants are shown by open circles.

Postural sway was assessed during the quiet standing trials. There was no effect of age group upon root‐mean‐square body angular velocity (0.36 ± 0.09 deg/s (young); 0.47 ± 0.34 deg/s (old); Mann–Whitney *U* = 167; *P* = 0.503; see Figure 3a).

Ankle stiffness was slightly higher for the smaller perturbations (5.60 ± 1.64 N m/deg (0.2 deg); 5.24 ± 1.36 N m/deg (0.8 deg); *F*_(1, 38) _= 10.6; *P* = 0.002; Figure 3b), but there was no effect of age group (5.03 ± 1.39 N m/deg (young); 5.68 ± 1.50 N m/deg (old) *F*_(1, 38) _= 1.91; *P* = 0.175). Relative stiffness was between 50% and 60% of gravitational toppling torque for both perturbation amplitudes and age groups (Figure 3c). Again, there was a significant effect of perturbation amplitude (56.1 ± 11.4% TT (0.2 deg); 53.0 ± 10.3% TT (0.8 deg); *F*_(1, 38) _= 13.25; *P *< 0.001) but no effect of age (53.0 ± 10.4% (young); 55.6 ± 10.7% (old); *F*_(1, 38) _= 0.58; *P* = 0.450).

### 3.2 Ankle stiffness and toppling torque

**FIGURE 4:**
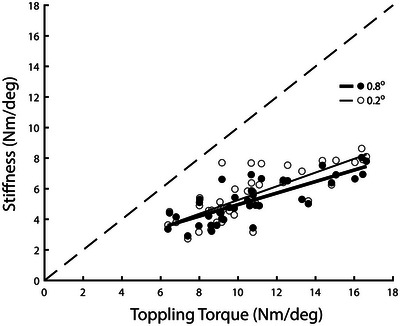
Relationship between ankle stiffness and gravitational toppling torque. Toppling torque was calculated from the ratio of ankle torque to body angle during a period of gentle voluntary antero‐posterior sway. Stiffness was calculated from platform perturbations of 0.2° and 0.8° amplitude. The dashed line depicts the line of unity. Stiffness is clearly less than toppling torque for all participants and perturbation amplitudes. Parameters for fitted regression lines are: *y* = 0.45*x* + 0.77 and *y* = 0.38*x* + 1.19 for 0.2° and 0.8° perturbations, respectively.

There was a significant relationship between ankle stiffness and toppling torque for both small and large perturbation amplitudes (Pearson's *r* = 0.78 and 0.79 for 0.2 and 0.8 amplitudes, respectively; *P* < 0.001 for both; Figure 4). However, ankle stiffness was consistently lower than gravitational toppling torque for all participants and perturbation amplitudes, as shown by all points lying below the line of unity in Figure 4.

### 3.3 Muscle movement

Individual examples of tracked fascicle length for the medial gastrocnemius are shown in Figure 1c,e during voluntary sway and quiet stance, respectively. Group mean values of the range of fascicle length were significantly greater during voluntary sway compared to quiet stance (3.3 ± 1.8 mm vs. 1.3 ± 0.6 mm; *F*_(1, 38) _= 68.0; *P *< 0.001). However, there was no effect of age upon the range of fascicle length (*F*_(1, 38) _= 2.69; *P* = 0.109). There was also no significant correlation between ankle stiffness and the range of fascicle motion (*r* ≤ 0.31; *P* ≥ 0.055).

**FIGURE 5:**
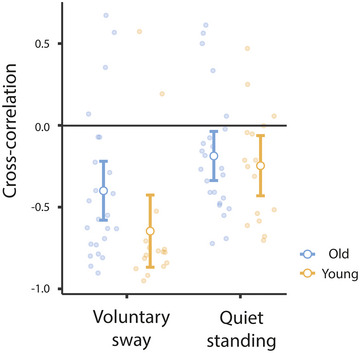
Muscle movement during different standing conditions. Values of peak cross‐correlations between calf muscle length and ankle angle are shown for voluntary sway and normal quiet stance. Open circles show mean values while filled circles depict individual values. Bars show 95% confidence limits of the mean.

To investigate the relationship between calf muscle motion and body sway, we calculated the peak of the cross‐correlation (CC) between the tracked fascicle length and antero‐posterior ankle angle (Figure 1d,f). Mean CC values were negative, indicating that the calf muscle generally shortened as the body swayed forwards. CC values were significantly lower during voluntary sway compared to quiet standing (Figure 5; mean values of −0.52 and −0.22, respectively; *F*_(1, 38) _= 15.44; *P *< 0.001). There was no effect of age group upon CC (*F*_(1, 38) _= 2.19; *P* = 0.147), nor any interaction between age and sway condition (*F*_(1, 38) _= 1.44; *P* = 0.237).

### 3.4 Relationship between ankle stiffness and muscle movement

**FIGURE 6:**
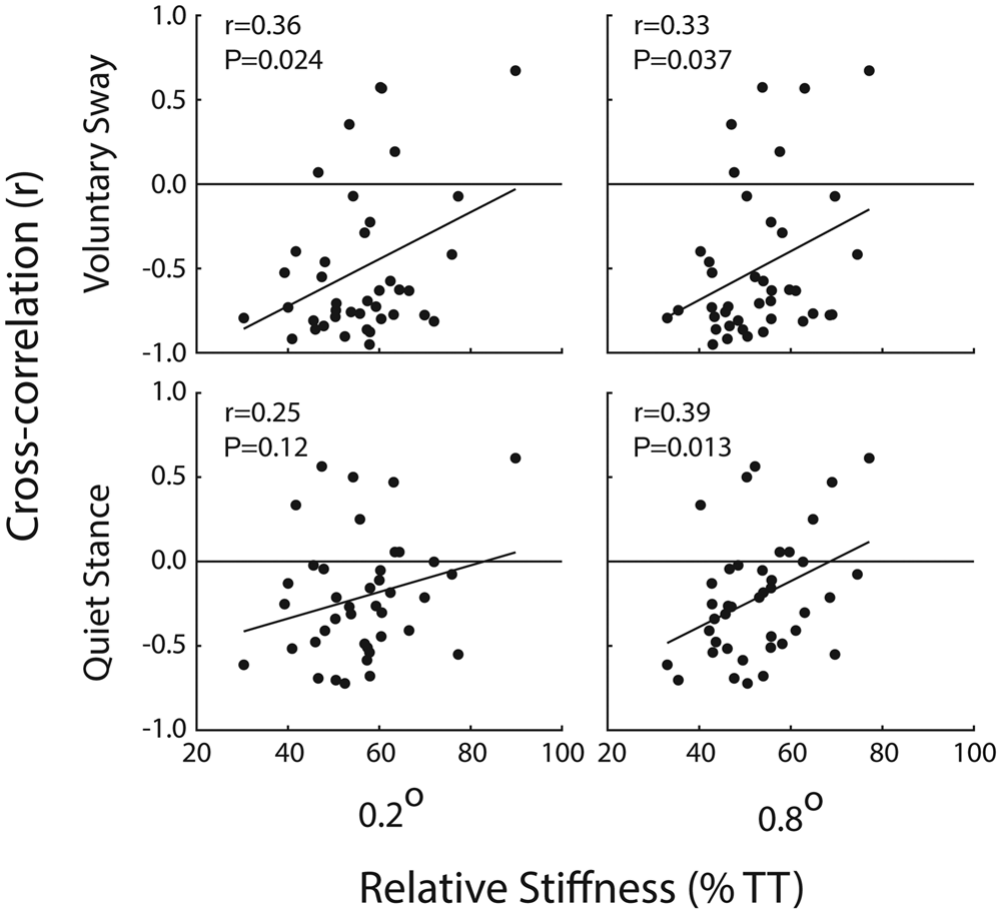
The relationship between ankle stiffness and muscle movement. Maximum values of cross‐correlations between ankle angle and muscle length are plotted against ankle stiffness, for both perturbation amplitudes, during both quiet stance and voluntary sway. Negative cross‐correlation values indicate that forward sway is associated with muscle shortening. Linear regression lines are shown, alongside *r* and *P* values.

Figure 6 shows cross‐correlation values plotted against relative ankle stiffness for each subject and condition. A positive correlation indicates that more compliant ankles are associated with ‘paradoxical’ muscle movement, that is, the calf muscle tends to shorten as the body sways forwards. All conditions exhibited a significant positive correlation (*r* ≥ 0.33; *P* ≤ 0.037), except for the 0.2° perturbation during quiet stance (*r* = 0.25; *P* = 0.119).

### 3.5 Correlation of ankle stiffness and muscle movement with postural sway

**FIGURE 7:**
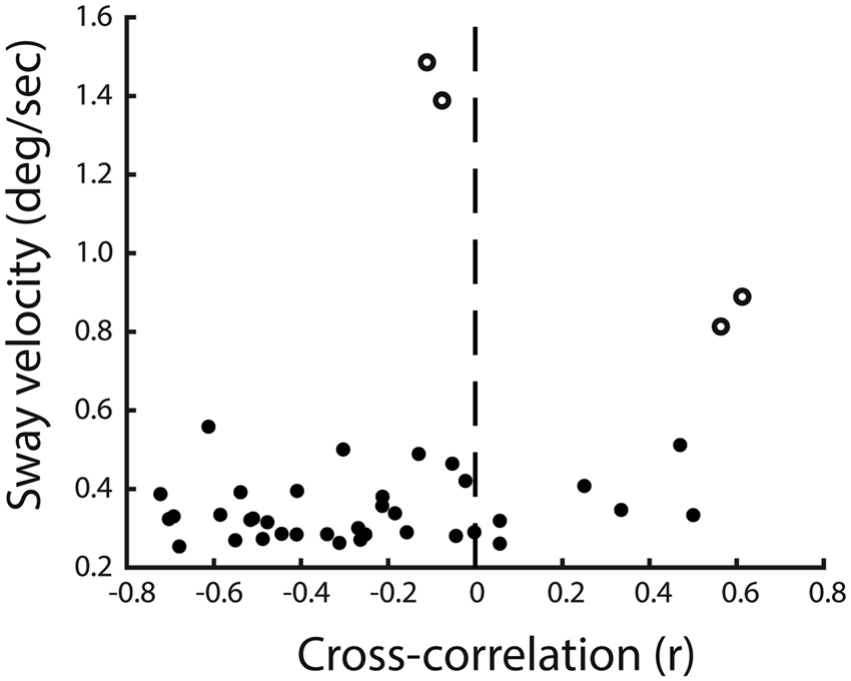
The relationship between sway and muscle movement. Cross‐correlations of muscle length versus ankle angle are plotted against RMS body angular velocity during quiet standing. Data are shown for all participants. Open circles depict outliers, that is, values exceeding 3rd + 1.5 interquartile range of sway velocity. After outliers are removed from the analysis there is no significant correlation (*r* = 0.18; *P* = 0.291).

Figure 7 shows the relationship between muscle‐sway cross‐correlation values during quiet stance, and RMS angular body velocity. Although there initially appears to be a *positive* correlation, four values of sway exceed the third interquartile range plus 1.5 × interquartile range, and so can be defined as clear outliers (see open circles in Figure 7). When these data points are ignored, the correlation is not significant (*r* = 0.18; *P* = 0.291).

Similarly, after removing these outliers there was no relationship between relative stiffness and RMS body sway angular velocity, either for 0.2° (*r* = −0.23, *P* = 0.170) or 0.8° perturbations (*r* = 0.017, *P* = 0.922).

## 4 DISCUSSION

In agreement with previous research, the calf muscle tended to *shorten* during forward sway (Fitzpatrick & Gandevia, 2005). This was shown by a negative cross‐correlation (CC) between calf muscle length and body angle, on average (−0.22). However, we also observed considerable individual variation in muscle movement. This variation was significantly related to ankle stiffness, with stiffer individuals exhibiting more ‘orthodox’ muscle motion (i.e., more positive CC values). However, neither ankle stiffness nor muscle movement was related to age or postural sway.

The tendency for paradoxical muscle movement during standing was first reported by Loram et al. (2004). Given that ankle stiffness is generally below the minimal level required to passively stabilise the body (Casadio et al., 2005; Loram & Lakie, 2002; Morasso & Sanguineti, 2002), they predicted that the calf muscles would shorten as the body sways forward, and indeed this was demonstrated for both the soleus and gastrocnemius muscles. They recorded calf muscle motion in three participants whose ankle stiffness varied by −0.1, +0.6 and +2 standard deviations from a mean value of 91% TT (as reported in Loram and Lakie, 2002). This higher mean value of stiffness compared to our values of 50%–60% TT is due to a smaller perturbation (0.055° vs. 0.2–0.8°) (Kearney & Hunter, 1982; Sakanaka et al., 2015). The most compliant subject showed the greatest paradoxical motion, with a CC value of approximately −0.12. The stiffest subject's value was ∼−0.75, with the intermediate at ∼−0.6. Here, we extend these observations by demonstrating a significant relationship between ankle stiffness and muscle movement in 40 participants. Variation between individuals ranged from −0.72 to +0.61 during quiet standing, and −0.95 to 0.67 during voluntary sway. Nevertheless, the majority of participants exhibited paradoxical movement. Only eight people (of 40) exhibited positive CC values indicative of orthodox muscle movement during quiet standing (see Figure 5). Hence, we can say that the tendency for the calf muscles to shorten during forward sway is prevalent, and very much associated with low ankle stiffness.

What is the source of ankle stiffness variation? During relatively low ankle torque levels maintained during quiet standing, the muscle is by far the stiffest element contributing to ankle stiffness, with the limiting factor being the Achilles tendon mechanics and foot anatomy (Jakubowski et al., 2023; Loram & Lakie, 2002). Any variation in the location of the Achilles insertion point, or the size and structure of the tendon or feet might be responsible for changes in joint stiffness. Since muscle and tendon size tend to co‐vary it is also possible that variation occurs secondarily to differences in strength and muscle mass. Such differences may be genetic, or related to training and exercise. Another source of variation may be subtle differences in standing posture and baseline ankle torque (Jakubowski et al., 2023; Sakanaka et al., 2015, 2021).

What are the functional consequences of variations in ankle stiffness? Low ankle stiffness results in calf muscle shortening during forward sway. The implication of this paradoxical muscle movement is that standing cannot be controlled by simple spinal stretch reflexes, since the muscle is not actually lengthening during forward sway. This implies that more complex active control is required, involving supraspinal structures. It might therefore be logical to assume that as the control task is made more complex by lower ankle stiffness, stability is compromised. Indeed, this has been observed in a manual balancing task involving an inverted pendulum and a spring (Lakie et al., 2003). As spring stiffness reduced from 186% to 58% TT, pendulum sway was approximately four times larger. However, here we did not observe any differences in postural sway related to ankle stiffness during standing. There was no relationship between postural sway and ankle stiffness or muscle movement. This suggests that, even if more compliant ankles do increase the complexity of the control task, the nervous system is more than capable of rising to this task. The nervous system can of course deal competently with different footwear and a wide range of supporting surfaces, all of which can artificially alter ankle stiffness, so this ability should not be unexpected. Most people can stand on one foot. This reduces total ankle stiffness by 50% by taking away the contribution of one leg. Furthermore, stable standing is possible on a sway‐referenced platform. In this case, the motion of the support surface is driven in real time by body position, that is, as the person sways forwards, the support surface simultaneously evokes a matching toes‐down tilt. By constraining ankle joint motion in this way, ankle stiffness is effectively reduced to zero, and yet standing is perfectly possible (Mergner et al., 2003). Nevertheless, it may be the case that when control mechanisms are impacted by neurological disease, stiffer ankles could be beneficial by minimising the load on the central nervous system.

In addition to requiring changes in motor output, a compliant ankle joint has obvious consequences for proprioception, since calf muscle length changes cannot be relied upon to encode body position in a clear and unambiguous manner. Given the established importance of lower leg muscle spindle information in the control of standing (Fitzpatrick et al., 1994), how does the nervous system compensate for this? One suggestion is that ankle joint position may be more faithfully encoded by spindles located in the tibialis anterior muscle (Di Giulio et al., 2009). Ultrasound recordings from TA exhibit length changes which are strongly positively correlated with body sway. So despite being largely passive during quiet stance, this muscle may play a more important role in the proprioceptive control of standing than the triceps surae.

Onambele et al. (2006) estimated the stiffness of the Achilles tendon using ultrasound, and found a significant decrease in stiffness with age which also correlated with postural sway. We did not find any effects of age on stiffness or muscle movement. This discrepancy may be due to methodological differences. Onambele et al. (2006) estimated stiffness by measuring displacement of the myotendinous junction in seated participants performing maximal voluntary contractions. In contrast, here we used direct mechanical perturbations applied during standing. Our technique assesses any structure in series which contributes to joint stiffness. This includes the foot and muscle, in addition to the Achilles tendon. Furthermore, the relatively low joint torque during quiet standing posture will likely result in different stiffness estimates to those seen during a maximum voluntary contraction. Ho and Bendrups (2002) found that older adults with a history of falling exhibited greater ankle stiffness than those who have not fallen. However, their perturbations were 8 s long, so inevitably will have measured reflex and/or voluntary responses, in addition to intrinsic stiffness. Nevertheless, it would be of interest to determine if intrinsic stiffness correlates with fall risk. Compared to the relatively fit and healthy cohort of adults studied here, this would require a larger cohort including those with a history of falls.

One potential limitation of our study is that subjects could theoretically have anticipated the arrival of the perturbation used to assess ankle stiffness, which occurred every 4–5 s. However, both the amplitude and direction of the perturbation was randomised. Furthermore, the mean values of stiffness were comparable to previously published estimates (Casadio et al., 2005).

In summary we have shown that there is considerable natural variation between individual's ankle stiffness. This stiffness determines their propensity for paradoxical muscle movement. However, neither parameter predicts postural sway.

## AUTHOR CONTRIBUTIONS

This research was undertaken in the laboratories of The School of Sport, Exercise & Rehabilitation Sciences at The University of Birmingham, UK. Raymond F. Reynolds obtained funding for the study. Raymond F. Reynolds, Anna M. Liedtke and Martin Lakie contributed to the conception and design of the experiment. Anna M. Liedtke performed the data collection. Anna M. Liedtke and Raymond F. Reynolds performed the data analysis. Raymond F. Reynolds drafted the manuscript. Raymond F. Reynolds, Anna M. Liedtke and Martin Lakie revised the manuscript critically for important intellectual content. All authors have read and approved the final manuscript and agree to be accountable for all aspects of the work in ensuring that questions related to the accuracy or integrity of any part of the work are appropriately investigated and resolved. All persons designated as authors qualify for authorship, and all those who qualify for authorship are listed.

## CONFLICT OF INTEREST

All authors report no conflicts of interest, commercial or otherwise.

## Supporting information



Spreadsheet containing the data reported in this study.

## Data Availability

A spreadsheet containing the data reported in this study is available under Supporting information.
